# Dietary specialization is conditionally associated with increased ant predation risk in a temperate forest caterpillar community

**DOI:** 10.1002/ece3.5662

**Published:** 2019-10-11

**Authors:** Michael S. Singer, Robert E. Clark, Emily R. Johnson, Isaac H. Lichter‐Marck, Kailen A. Mooney, Kenneth D. Whitney

**Affiliations:** ^1^ Department of Biology Wesleyan University Middletown CT USA; ^2^ Department of Ecology and Evolutionary Biology University of California at Irvine Irvine CA USA; ^3^ Department of Biological Sciences University of New Mexico Albuquerque NM USA

**Keywords:** antipredator defense, enemy‐free space, host specificity, insect herbivores, polyphagy, tritrophic interactions

## Abstract

The enemy‐free space hypothesis (EFSH) contends that generalist predators select for dietary specialization in insect herbivores. At a community level, the EFSH predicts that dietary specialization reduces predation risk, and this pattern has been found in several studies addressing the impact of individual predator taxa or guilds. However, predation at a community level is also subject to combinatorial effects of multiple‐predator types, raising the question of how so‐called multiple‐predator effects relate to dietary specialization in insect herbivores. Here, we test the EFSH with a field experiment quantifying ant predation risk to insect herbivores (caterpillars) with and without the combined predation effects of birds. Assessing a community of 20 caterpillar species, we use model selection in a phylogenetic comparative framework to identify the caterpillar traits that best predict the risk of ant predation. A caterpillar species' abundance, dietary specialization, and behavioral defenses were important predictors of its ant predation risk. Abundant caterpillar species had increased risk of ant predation irrespective of bird predation. Caterpillar species with broad diet breadth and behavioral responsiveness to attack had reduced ant predation risk, but these ant effects only occurred when birds also had access to the caterpillar community. These findings suggest that ant predation of caterpillar species is density‐ or frequency‐dependent, that ants and birds may impose countervailing selection on dietary specialization within the same herbivore community, and that contingent effects of multiple predators may generate behaviorally mediated life‐history trade‐offs associated with herbivore diet breadth.

## Graphical abstract

This study is an experimental test of the hypothesis that dietary specialization of insect herbivores reduces their predation risk. Unexpectedly, ant predation of caterpillars increased with dietary specialization and depended on bird predation. In this community, dietary specialization was associated with reduced behavioral defenses, which predicted ant predation risk in a way that was also contingent upon bird predation.

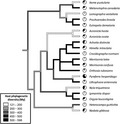

## 1 INTRODUCTION

The “enemy‐free space hypothesis” (EFSH) holds that the evolution of dietary specialization of small herbivorous arthropods is selected by fitness benefits arising from the use of specific host plants for defense or refuge from generalist predators (Bernays & Graham, 1988). Several studies support the community‐level prediction of the EFSH that dietary specialization of insect herbivores reduces their risk of predation by generalist predators (e.g., Bernays, 1988, 1989; Bernays & Cornelius, 1989; Dyer, 1995, 1997; Dyer & Floyd, 1993; Singer et al., 2014). The most established mechanism for superior antipredator defenses of host‐specific herbivores is their advantage over dietary generalists at sequestering secondary metabolites specific to their host plants (Dyer, 1995; Zvereva & Kozlov, 2016), thus gaining a measure of enemy‐free space (Jeffries & Lawton, 1984) from generalist predators in their communities. In addition, recent work shows that dietary specialization is associated with superior camouflage and reduced bird predation risk (Singer et al., 2014).

However, predation at a community level is also subject to combinatorial effects of multiple‐predator types (Sih, Englund, & Wooster, 1998), and a multiple‐predator perspective has mostly been lacking in tests of the EFSH. A recent study of bird and ant predation on the suppression of forest caterpillars in Connecticut showed evidence for functional complementarity between these two predator groups (Singer, Johnson, Lichter‐Marck, Clark, & Mooney, 2017). In this case, ant predation was stronger on specialist caterpillars (contrary to the EFSH), while bird predation suppressed generalist caterpillars in support of previous findings (Singer et al., 2014; Zvereva & Kozlov, 2016). The factorial exclusion of birds and ants in Singer et al. (2017) also revealed a predator–predator interaction that had not been predicted by prior theory or evidence: predation effects of ants on dietary specialist caterpillars were detectable only when birds were not excluded. Although unknown, some possible mechanisms underlying this predator–predator interaction include: (a) bird effects on caterpillar behavior that specifically enhance ant predation risk of dietary specialist caterpillars, (b) bird predation of invertebrate predators, such as spiders, thus indirectly changing ant–caterpillar interactions, and (c) exploitation competition between birds and ants, thus relegating ant predation to caterpillar species avoided by birds (i.e., dietary specialist caterpillars) (Singer et al., 2017). Regardless of the mechanism, bird and ant partitioning of caterpillars by diet breadth and body size has the potential to generate life‐history trade‐offs among caterpillar species rather than merely select for dietary specialization as the EFSH proposes.

Because the patterns of ant predation on caterpillars found in Singer et al. (2017) were not anticipated by prior theory or evidence, we use a new methodology and additional data to scrutinize them from an evolutionary ecology perspective. To address the effect of herbivore diet breadth on ant predation, we employ a phylogenetic comparative analysis of different caterpillar species and their diet breadth, estimated as host phylogenetic diversity (see Singer et al., 2014). Unlike Singer et al. (2017), which categorically defined dietary specialist and generalist caterpillars without disaggregating the species in those categories, the phylogenetic comparative analysis used here and elsewhere (Singer et al., 2014) can distinguish phylogenetically widespread patterns in ecological communities from ecological patterns owing to strong effects of one or few dominant species. To address combinatorial effects of ant and bird predation shown in Singer et al. (2017), we used the phylogenetic comparative approach to analyze effects of dietary specialization of herbivores to ant predation occurring with and without combined effects of bird predation.

Because herbivore diet breadth per se is not an antipredator trait, we consider several other life‐history traits of caterpillar species that might underlie or act in concert with diet breadth. These traits, which include behavioral defenses, body size, and abundance, were chosen because they have been found or hypothesized to both influence caterpillar predation risk and correlate with diet breadth (Dyer, 1995; Greeney, Dyer, & Smilanich, 2012; Lichter‐Marck, Wylde, Aaron, Oliver, & Singer, 2015; Montllor & Bernays, 1993; Remmel, Davidson, & Tammaru, 2011; Singer et al., 2014). As such, there is the potential to identify syndromes of defensive traits involved in life‐history trade‐offs among caterpillar species in the same community.

We focus on the role of behavioral defenses, which have received limited attention in studies of the EFSH despite some hints that they might be important. The palatability experiments used in previous EFSH studies eliminated or reduced possible effects of behavioral defenses by using freshly killed caterpillars (e.g., Bernays & Cornelius, 1989) or live caterpillars removed from their host plants and experimentally placed near ant nests or foraging trails (e.g., Dyer, 1995, 1997; Dyer & Floyd, 1993). Consequently, these palatability experiments successfully tested one specific mechanism of the EFSH, that is, dietary specialization imparts EFS via superior co‐opting of plant allelochemicals, while offering limited opportunities to test alternative mechanisms. In addition to primary defenses (i.e., those that prevent predator attacks), such as warning signals and camouflage, prey species frequently deploy secondary defenses (i.e., those that enable prey to survive attack), such as behavioral responses to attack (Gross, 1993). Previous research suggests that unlike chemical defenses (Zvereva & Kozlov, 2016), behavioral defenses of caterpillars are most effective against invertebrate predators in particular (reviewed in Greeney et al., 2012; Montllor & Bernays, 1993). Based on evidence that dietary generalist caterpillars and tortoise beetle larvae exhibit more behavioral defenses than specialists do (Bernays, 1988; Coley, Bateman, & Kursar, 2006; Vencl, Nogueira‐de‐Sa, Allen, Windsor, & Futuyma, 2005), we hypothesize that behavioral defenses associated with dietary generalization might offset or trade off with unpalatability associated with dietary specialization. To address this hypothesis, we measure ant predation risk in situ coupled with laboratory behavioral assays for each caterpillar species. We use these data to test if the expression of behavioral defenses mediates the putative relationship between ant predation risk and diet breadth of herbivores.

In summary, this study attempts to resolve conflicting evidence from studies testing ant predation on dietary specialist and generalist caterpillars by combining a comparative evolutionary ecology approach (Bernays & Cornelius, 1989; Dyer, 1995, 1997; Dyer & Floyd, 1993; Singer et al., 2014; Vencl et al., 2005) with a multiple‐predator perspective (Singer et al., 2017). We ask whether ant predation biased toward dietary specialist herbivores and contingency on bird predation are community‐wide patterns that oppose the community‐level prediction of the EFSH. Or, are these patterns driven by dominant, outlier herbivore species, with most species conforming to the EFSH prediction of reduced ant predation on dietary specialists? How do these patterns relate to other putatively important herbivore traits, such as behavioral defenses, mobility, body size, and abundance, which all may correlate with diet breadth? In particular, we address the hypothesis that dietary specialization trades off with the strength of behavioral defenses in the herbivore community.

## 2 METHODS

### 2.1 Study system

We studied ant predation of an assemblage of externally feeding caterpillars (larval Lepidoptera) naturally occurring on eight taxa of native deciduous trees. These tree species are characteristic of oak‐hickory‐beech‐maple upland forest in the northeastern coastal forest ecoregion (Olson et al., 2001) of the USA, and include *Acer rubrum* (red maple, Sapindaceae), *Betula lenta* (black birch, Betulaceae), *Carya* spp. (hickory, Juglandaceae) in the Eucarya group (*C. ovata, C. glabra, C. tomentosa*), *Fagus grandifolia* (American beech, Fagaceae), *Hamamelis virginiana* (witch hazel, Hamamelidaceae), *Prunus serotina* (black cherry, Rosaceae), *Quercus alba* (white oak, Fagaceae), and *Quercus rubra* (red oak, Fagaceae). The caterpillar assemblage included at least 70 spp. in 10 families, with numerous dietary generalist species that eat most or all of the tree taxa studied, as well as dietary specialist species that feed only on tree species within a single family (Singer, Farkas, Skorik, & Mooney, 2012; Singer et al., 2014). The most important ant predators of caterpillars in this community are *Formica neogagates*, *Camponotus chromaiodes*, and *Camponotus pennsylvanicus* (Clark, Farkas, Lichter‐Marck, Johnson, & Singer, 2016). As in previous studies of this community that focus on bird predation of caterpillars (Lichter‐Marck et al., 2015; Singer et al., 2012, 2014), we conducted fieldwork at three sites (Cockaponset State Forest, Haddam; Hurd State Park, East Hampton; Millers Pond State Park, Durham (all in Middlesex County, CT), each with six spatially replicated experimental blocks (ca. 1 ha in size) containing each tree species.

### 2.2 Ant‐exclusion field experiment

To test the effect of ants on the caterpillar community, we performed an ant‐exclusion field experiment at each of the field sites described above in May–July of 2011, 2012, and 2016. Ant‐exclusion branches (*N* = 563) had a 6–8‐cm‐wide ring of sticky resin (Tanglefoot®, Contech Enterprises) at the base and were isolated from the rest of the canopy by choice of branch or by pruning nonexperimental branches if necessary. The control branches (*N* = 563) were not manipulated with resin. To minimize other differences among experimental branches within each replicate, we applied treatments and controls to the same individual tree whenever possible, or used spatially proximate conspecific trees of similar size and light exposure, and used branches of similar overall size, height, and leaf number. We assigned treatments to individual branches within a replicate in a haphazard manner.

In 2011 only, the ant‐exclusion treatment was crossed with a bird‐exclusion treatment, as part of a factorial manipulation of bird and ant predation effects on caterpillars (described in detail in Singer et al., 2017). We excluded birds with nylon mesh bags that enveloped tree branches; the mesh size of the netting (13 or 20 mm) was large enough to allow access by invertebrates while excluding birds. Bird‐exclusion treatments were applied at the same time as the ant‐exclusion treatments. When analyses showed that some of the ant effects on caterpillars depended on bird exclusion (Singer et al., 2017) and the analyses for the present study demanded more data from dietary specialist caterpillar species from bird‐exclusion branches (which were lacking in 2012), we set up an additional trial of the ant‐exclusion experiment with all experimental (treatment and control) branches outfitted with bird‐exclusion bags (May–July 2016) using the same methods as we used in 2011. This additional trial was more modest in scope than the ant‐exclusion experiment trials of 2011 and 2012, as it included only a subset of host plant species (*H. virginiana, P. serotina,* and *Q. alba*) that host the focal dietary specialist caterpillar species for which additional data were needed.

We set up the predator‐exclusion treatments during May of each year and sampled during June and July as follows. Beginning in the second week of May, we located each experimental branch and knocked it with a stick (beating) repeatedly in two 5‐s bouts separated by ca. 5 s to dislodge arthropods onto a sheet held below. Beating each branch served to remove any ants prior to applying predator‐exclusion treatments and was applied to all branches regardless of the experimental treatment. Herbivores dislodged by beating were returned to their respective branches. Then we applied the experimental treatment or merely labeled the branch in the case of control branches. In each of the three successive weeks, we set up two blocks separated by at least 1,000 m at each of the three sites. We began two rounds of sampling in the first week of June following the same sequence of setup, such that each experimental branch was sampled both at three weeks and six weeks after setup. We sampled during daylight hours (0900–1600 hr) by beating branches, collecting ants and caterpillars, and bringing all ants and a subset of caterpillars (see below) back to the laboratory for measurement, rearing, and identification. We recorded the number of all ants and caterpillars found on each branch. However, we replaced, rather than collected, caterpillars under ca. 1 cm in length because of the low likelihood that they would survive collection and transport from field to lab (MSS, personal observations). Therefore, species determinations could only be made for the set of caterpillars ≥1 cm in length. For each caterpillar collected in 2011 and 2012, we measured its length to the nearest mm within 24 hr of collection. Each caterpillar was then placed in a separate container and reared on leaves collected from the tree species on which it was found. Most caterpillars could be identified from field guides (Wagner, 2005; Wagner, Ferguson, McCabe, & Reardon, 2001; Wagner, Schweitzer, Sullivan, & Reardon, 2011), but determination of some species required rearing them to adults. Knowing the species identities of each caterpillar enabled us to determine the set of plant species eaten by each caterpillar species based on a multiyear compilation (2004–2016) of host plant records associated with each caterpillar species in this community (Singer et al., 2014; see below).

We measured ant predation risk as the effect of ants on the population density of each caterpillar species. Effect sizes of ants on each caterpillar species were calculated as log response ratios (LRR; Hedges, Gurevitch, & Curtis, 1999) according to the formula LRR_ant_ = ln(control population density/exclusion population density). Thus, values of LRR_ant_ are increasingly negative with increasing ant suppression of caterpillar density, and we interpret low values of LRR_ant_ to indicate high ant predation risk. Because this metric of predation risk is indirect, we consider alternative interpretations of LRR_ant_ as well (see below). Summed over 2011 and 2012, 20 caterpillar species were sampled >10 times (*N* = 11–277) from the total of 1,054 sampled tree branches or saplings (each sampled twice during the season of each year) (Table S1). We used the data from 2016 (*N* = 64 branches or saplings) to supplement sample sizes of some of these 20 focal caterpillar species. Limited sample sizes of most caterpillar species necessitated that counts of each species be pooled across all years to get a single population density value for each treatment. In light of small sample sizes for some caterpillar species, we consider that values of LRR_ant_ based upon small sample sizes are subject to bias, especially as numerator or denominator values approach zero (Lajeunesse, 2015). The procedures proposed to correct for this bias require the calculation of LRR variances (Lajeunesse, 2015). Because several caterpillar species were rare and we calculated a single LRR_ant_ value for each species in each analysis (see Section 2.5), our approach does not allow us to calculate corrected LRR_ant_ values. However, while some of our uncorrected LRR_ant_ values may be positively or negatively biased, this bias should not influence the direction or magnitude of the correlations between LRR_ant_ and caterpillar species traits described below (see Section 2.5).

### 2.3 Caterpillar traits

To test the EFSH, the putative role of antipredator behavior in mediating its dynamics, and other factors of hypothesized importance, we assessed the following traits of caterpillars as possible predictors of ant predation risk (via LRR_ant_).

#### 2.3.1 Diet breadth

We quantified the variation in diet breadth among caterpillar species as host phylodiversity (HPD; Poulin, Krasnov, & Mouillot, 2011), the aggregate phylogenetic distance in millions of years between host plants used by each caterpillar species. Calculation of HPD for these species is detailed in Singer et al. (2014). Briefly, the phylogenetic topology of the eight host angiosperm species and their most recent common ancestor was estimated from the Davies et al. (2004) supertree via the Phylomatic program (Webb & Donoghue, 2005) (Figure S1). Node ages (mya) were obtained from Wikstrom, Savolainen, and Chase (2001) (assuming the ACCTRAN optimization). The single remaining undated node (that connecting *Quercus rubra* to *Q. alba*) was estimated to be equidistant between the tips and the *Fagus*/*Quercus* split (34 mya), that is, at 17 mya. Branch lengths (mya) were then calculated from the node ages. Following Poulin et al. (2011), host phylodiversity (HPD) for each caterpillar species was calculated as the total branch length (in millions of years) linking its host species along this phylogenetic tree; this was implemented using the Phylogenetic Diversity (pd) command in the Picante package (Kembel et al., 2010) in R (R Core Team, 2015).

#### 2.3.2 Frequency of behavioral response (FBR) to simulated predator attack

To systematically compare putative antipredator behaviors among caterpillar species, we conducted a simulated predation assay (sensu Sendoya & Oliveira, 2017) in the laboratory with all the caterpillars collected in the ant‐exclusion experiment during 2011 and 2012. Within 24 hr of being collected from the field, each caterpillar was removed from its collection vial by handling the piece of its host plant upon which it was attached. After it was transferred from its vial to the lab bench, we waited until each caterpillar came to rest before slowly raising the piece of host plant from the bench and pinching the caterpillar on the posterior part of the body with blunt forceps (featherweight forceps, BioQuip) to mimic attack from a biting predator. We recorded all expressed behaviors for ca.10 s, and we grouped these into seven categories: thrash, bite, regurgitate, hold on, drop, evade, and still (Lichter‐Marck et al., 2015). We consolidated the first six of these categories into a "response" category, whereas "still" was considered as the lack of a behavioral response. Then we calculated the frequency of behavioral response (FBR) for each caterpillar species with five or more records as the number of individuals that exhibited any behavioral response divided by the total number of individuals tested.

#### 2.3.3 Frequency of fleeing from ants (FF)

To better understand the role of caterpillar antipredator behavior in situ (while herbivores are situated on their host plants), we conducted a behavioral assay in which staged encounters between caterpillars and ants were observed and recorded (sensu Sendoya & Oliveira, 2017). Staged encounters occurred on potted saplings (0.5–1.5 m tall) of four tree species, *Acer rubrum* (red maple), *Hamamelis virginiana* (witch hazel), *Prunus serotina* (black cherry), and *Q. alba* (white oak), which were chosen because they host most of the dietary specialist and generalist caterpillars analyzed in the ant‐exclusion field experiment. In each trial, field‐collected caterpillars (mean 2.27 ± 0.70 *SD* cm in length, *N* = 331) of several specialist and generalist species (1–4 individuals) were placed on upper canopy shoots of a potted sapling, which in turn was placed 1–2 m from the nest entrance of a carpenter ant colony (*C. pennsylvanicus*) on the campus of Wesleyan University. After letting the caterpillars habituate to the sapling (ca. 3–5 min), several (3–5) worker ants were gathered from foraging trails and placed on branches near the experimental caterpillars, and observations were made for 60 min per trial. During each trial, the behavioral responses of each caterpillar physically contacted by one or more ants were recorded. Each individual caterpillar was used once in the experiment, and trials occurred over 24 days (May–July, 2013) during daylight hours (0800–1900 hr). The suite of behavioral responses observed was the same as the responses described in the frequency of behavioral response (FBR) assay described above, with the addition of "rappelling on a silk line." We scored the frequency of fleeing from ants (FF) for each caterpillar species with more than five records as the number of individuals that responded with drop, evade, or rappelling on a silk line divided by the total number of observations of that species (Table S1). Because this experiment had smaller sample sizes and a subset of the caterpillar species used in the FBR assay described above, we do not use its data to predict the risk of ant predation (LRR_ant_). Rather, we use the data to test the relationship between escape behavior (FF) and caterpillar diet breadth (HPD). In light of unexpected evidence from the ant‐exclusion experiment that dietary specialization increased the risk of ant predation (see Section 3), we test the alternative possibility that our measure of high risk of ant predation (low values of LRR_ant_) for dietary specialist caterpillars (low HPD) does not actually indicate predation risk, but instead indicates that dietary specialists were more likely than generalists to escape, resulting in disappearance from the experimental branch. This alternative hypothesis, which is consistent with the EFSH, predicts that dietary specialists should have higher probabilities than generalists of fleeing from ants (a negative correlation between the FF and HPD).

#### 2.3.4 Mobility

We hypothesized that a caterpillar species' overall mobility might predict its ability to avoid ant predation. To address this possibility, we compiled measures of each caterpillar species' frequency of directed movement (locomotion or rappelling on a silk line) from direct observations of unmanipulated caterpillars made during 2004–2007 and 2013 at the same field sites used for the ant‐exclusion experiment. Observers searched the focal tree species for caterpillars both systematically and opportunistically (Farkas & Singer, 2013). When a caterpillar was located, its behavior at that instant (resting, feeding, locomoting, and rappelling on a silk line) as well as its identity was recorded. To compare these individual observations as species traits, we calculated the mobility of each caterpillar species as the number of occurrences of locomoting + rappelling on a silk line divided by the total number of observations of that species (Table S1).

#### 2.3.5 Body size

Previous work (Remmel et al., 2011; Singer et al., 2017) suggested that small‐bodied caterpillars would be especially susceptible to ant predation and that body size could be related to diet breadth (Davis, Ounap, Javois, Gerhold, & Tammaru, 2012). To estimate body size, we measured the body length of each caterpillar (when its body was extended while at rest) sampled from the ant‐exclusion experiment. We calculated the mean body length recorded for each caterpillar species, calculated from individuals in the ant‐exclusion treatment only (2011 and 2012) (Table S1). We measured individuals with digital calipers (rounded to the nearest millimeter).

#### 2.3.6 Abundance

We considered variation in abundance among caterpillar species to test for the possibility that ant predation risk would vary nonlinearly in relation to prey species abundance due to density‐ or frequency‐dependent predation behavior, as seen in other predators (e.g., Kuang & Chesson, 2010; Royama, 1970). We included in analyses the sample size of each caterpillar species with >10 records from the ant‐exclusion experiment (2011 and 2012) as a measure of total abundance. Because the range in sample size varied from 11 to 277 (Table S1), sample size was log‐transformed to meet assumptions of normality in statistical analyses. We omitted the supplemental sample size data (2016) from our estimate of abundance because these abundance data, sampled from only three tree species, were biased with respect to the data from 2011 and 2012. Therefore, caterpillar species abundances from 2016 are not comparable to those from 2011 and 2012.

### 2.4 Phylogenetically independent contrasts

In our comparative analyses, we accounted for phylogenetic nonindependence of our samples (caterpillar species) via phylogenetically independent contrasts (PICs; Felsenstein, 1985). A composite phylogeny for the 20 caterpillar species was constructed in Mesquite v. 3.01 (Maddison & Maddison, 2014) based on molecular phylogenetic trees reported in Regier et al. (2009), Zahiri et al. (2011, 2012), and Sihvonen et al. (2011). Expert opinion (David L. Wagner, U. Connecticut, pers. comm.) was then used to resolve polytomies in three small clades. Further details on phylogeny construction are given in Singer et al. (2014), which reports a larger phylogeny of 41 species that includes the 20 species discussed here. Given uncertainty in the placement of the family Nolidae, we considered two alternative topologies in all downstream analyses (see Figures S1 and S2). Because branch lengths were unknown, we tested branch lengths corresponding to all branches = 1.0 and Grafen's arbitrary lengths (generated using the R package *ape* v. 3.4, Paradis, Claude, & Strimmer, 2004). We present results only for all branches = 1.0 as diagnostics indicated that they provided the best fit. We then calculated standardized PICs using the pic command in *ape* v. 3.4 (Paradis et al., 2004).

### 2.5 Statistical analysis

We used an information‐theoretic model selection procedure (Anderson, 2008) to determine the best statistical model to explain variation in ant predation risk among caterpillar species (LRR_ant_). Phylogenetically independent contrasts (PICs) for the five predictor variables (host phylogenetic diversity [HPD], frequency of behavioral response [FBR], mobility, body size, and abundance) were each standardized to mean 0 and standard deviation of 1. We used the MuMIn package (Barton, 2015) in R to evaluate GLM models. Because we considered our sample sizes (*N* = 20 caterpillar species) insufficient to exhaustively test all possible combinations of our five predictors, we first performed an exploratory analysis (following Anderson, 2008, pp. 118–120) by estimating Relative Variable Importance (RVI: for a given variable, the sum of Akaike model weights (*w_i_*) for all models containing it; Anderson, 2008) using a set of 32 models containing all possible combinations of the five predictors but excluding interactions between them. The three highest‐ranked predictors identified were then used in downstream analyses (note that these were chosen by rank, not by their relationship to any arbitrary RVI cutoff value). This procedure resulted in consideration of eight models analyzing ant predation risk with phylogenetically independent contrasts (picLRR_ant_), consisting of the model picLRR_ant_ = picHPD picFBR picAbundance, all six nested models, and a null.

We then examined alternative estimates of picLRR_ant_ for the set of experimental branches with bird access (with bird effects) versus those excluding birds (without bird effects). The estimate of picLRR_ant_ with bird effects was calculated for each caterpillar species with five or more records across all bird‐accessible branches, whereas the estimate of picLRR_ant_ without bird effects was calculated for each caterpillar species with five or more records across all bird‐exclusion branches (2011, 2012, 2016). For each predictor (HPD, FBR, or abundance), we ran the model picLRR_ant_ = picPredictor +Bird Treatment + picPredictor x Bird Treatment. These analyses were followed by regressions of picLRR_ant_ with and without bird effects on picHPD and picFBR, which showed significant interactions with Bird Treatment in the first set of models. Finally, we ran the model picLRR_ant_ with bird effects = picHPD +picFBR to analyze the relative contributions of diet breadth (HPD) and frequency of behavioral response (FBR) to ant predation risk in the presence of bird effects.

To test if dietary specialization and behavioral responsiveness covaried among caterpillar species, we used a Pearson's product‐moment correlation to examine the relationship between the frequency of behavioral responses (picFBR) and diet breadth (picHPD) (*N* = 20 species).

To test the prediction that dietary specialists should have higher probabilities than generalists of fleeing from ants (a negative correlation between the FF and HPD), we used a Pearson's product‐moment correlation to test the relationship between the frequency of fleeing from ants (picFF) and diet breadth (picHPD) (*N* = 16 species).

Models were constrained through the intercept (0,0) as is appropriate for PICs (Garland, Harvey, & Ives, 1992). Because of limited sample sizes, interactions between predictor variables were not considered. Model selection followed AICc rankings. Parameter estimates (*β*s) were averaged across all models in which each parameter appeared, weighted by Akaike model weight (*w_i_*) (Anderson, 2008).

## 3 RESULTS

### 3.1 Predictors of ant predation risk

The effect of exposure to ant predators varied among the 20 caterpillar species, with ant predation risk (LRR_ant_) ranging from –1.05 (caterpillar density reduced from 0.0078 to 0.0027 per m^2^ foliage, *Pyreferra hesperidago*) to 0.82 (caterpillar density increased from 0.010 to 0.023 per m^2^ foliage, *Crocigrapha normani*). Across all possible 32 PIC models predicting ant predation risk (LRR_ant_), the caterpillar traits with the highest relative variable importance (RVI) were abundance, frequency of behavioral response (FBR), and diet breadth (HPD); this result was consistent using either phylogenetic tree 1 or 2 (Figures S1 and S2, Table 1). Because of their low RVI values, average body size and mobility were not considered in further analyses. Among the eight PIC models based on all possible combinations of abundance, FBR, and HPD, the best model (lowest AICc) included abundance and HPD as the predictors using phylogenetic tree 1 and abundance and FBR as the predictors using phylogenetic tree 2 (Table 2). However, these best models could not be distinguished statistically from rival models that included all three predictors, the alternative 2‐predictor model in each case, or a model with FBR as the sole predictor (i.e., Δ AICc < 2; Table 2).

**Table 1:** Predictors of the risk of ant predation across caterpillar species

Trait	Relative variable Importance Tree1	Relative variable Importance Tree2	Sum of RVI across trees
**Abundance**	0.69	0.70	1.39
**FBR**	0.56	0.70	1.26
**HPD**	0.63	0.60	1.23
Length	0.46	0.45	0.91
Mobility	0.20	0.19	0.39

Note

Relative Variable Importance (RVI) for five traits, based on analysis of the full model set (32 models), each using PICs to test the association between traits and LRR_ant_ (caterpillar response to ant predation). FBR refers to the frequency of behavioral response by caterpillars subjected to simulated predator attack, and HPD refers to host phylodiversity (our metric of caterpillar diet breadth). Results are presented for two alternate tree topologies differing in their placement of the Nolidae (see Section 2 and Figures S1 and S2). RVI is the sum of the Akaike model weights of each model in which a trait appears. The top three traits chosen for further evaluation in multivariate analyses are in boldface type. Note that mobility = "freq walking + silking." *N* = 20 caterpillar species.

**Table 2:** Comparison of phylogenetically independent contrast models explaining LRR_ant_ (caterpillar risk of ant predation) as a function of caterpillar traits

Model	AICc	∆AICc	Log likelihood	*w_i_*	Adj *R* ^2^
Tree1					
Abun + HPD	10.67	0.00	−1.53	0.27	.79
Abun + FBR	11.20	0.53	−1.80	0.21	.75
Abun + FBR+HPD	11.90	1.23	−0.52	0.14	.93
FBR	11.94	1.27	−3.59	0.14	.44
HPD	13.06	2.39	−4.16	0.08	.34
FBR + HPD	13.81	3.15	−3.11	0.06	.53
Abun	13.90	3.23	−4.57	0.05	.25
Null	13.99	3.32	−5.88	0.05	−.03
Tree2					
Abun + FBR	10.40	0.00	−1.40	0.25	.81
Abun + FBR+HPD	10.85	0.45	0.00	0.20	1.00
Abun + HPD	10.95	0.55	−1.68	0.19	.77
FBR	11.47	1.07	−3.36	0.15	.50
FBR + HPD	13.24	2.84	−2.82	0.06	.59
HPD	13.26	2.85	−4.25	0.06	.34
Null	14.02	3.62	−5.89	0.04	−.01
Abun	14.02	3.62	−4.64	0.04	.26

Note

Results are presented for two alternative tree topologies of the Lepidoptera phylogeny differing in their placement of the Nolidae (Tree 1, Tree 2; see Section 2 and Figures S1 and S2).

Abbreviations: Abun, abundance; FBR, frequency of behavioral response; HPD, host phylodiversity; *w_i_*, Akaike model weight.

**Figure 1:**
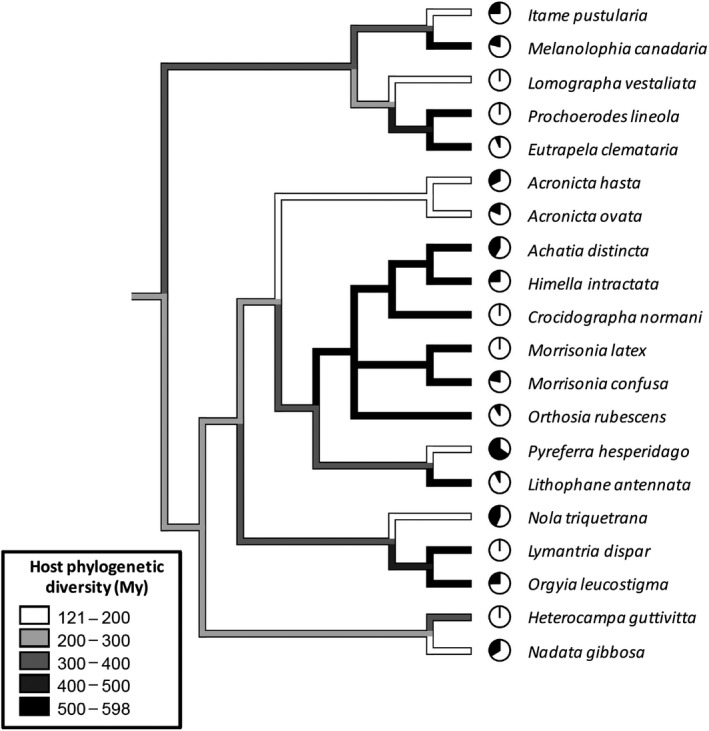
A reconstruction of caterpillar diet breadth (host phylodiversity, HPD, in millions of years) mapped onto the caterpillar phylogeny (tree 1; see Section 2 for details). HPD represents the aggregate phylogenetic distance among hosts, here derived from branch lengths on a dated phylogeny. Higher values of HPD indicate more generalized diets. The black area of each pie chart at the branch tips shows the magnitude of the ant predation effect [percentage density reduction, calculated by exponentiating the log response ratio, ln(control caterpillar density/ant‐exclusion caterpillar density)] for each caterpillar species in the field experiment

For this group of models, the predictor variables had consistent relationships with ant predation risk (LRR_ant_) regardless of which phylogenetic tree topology was used (Table 3). Abundance was negatively associated with LRR_ant_, indicating that relatively abundant caterpillar species experienced the strongest reductions in density from ants. By contrast, frequency of behavioral response (FBR) was positively associated with LRR_ant_, suggesting that caterpillar behavioral responses observed in the lab via simulated attack served a defensive function against ants in nature. Similarly, caterpillar diet breadth (HPD) was positively associated with LRR_ant_, indicating that broader diet breadth reduced a caterpillar species' risk of ant predation (Figure 1). RVI values consistently indicated that abundance was the most important predictor, but analyses using the alternative phylogenetic trees differed in terms of the relative importance of FBR and HPD, either equal in importance (tree 1) or with FBR more important (tree 2) (Table 3).

**Table 3:** Effects of caterpillar traits on the risk of ant predation

Trait	Standardized *β*	95% CI lower	95% CI upper	Relative variable importance
Tree1				
Abundance	−0.42	−0.84	0.01	0.67
HPD	0.39	−0.06	0.85	0.55
FBR	0.41	−0.06	0.87	0.55
Tree2				
Abundance	−0.41	−0.819	−0.0003	0.69
FBR	0.44	−0.002	0.891	0.66
HPD	0.38	−0.084	0.839	0.52

Note

Model‐averaged parameter estimates based on the set of models reported in Table 2. Positive *β*s indicate that risk decreases with an increase in the trait.

Abbreviations: FBR, frequency of behavioral response; HPD, host phylodiversity.

### 3.2 Ant predation risk in combination with bird predation

**Figure 2:**
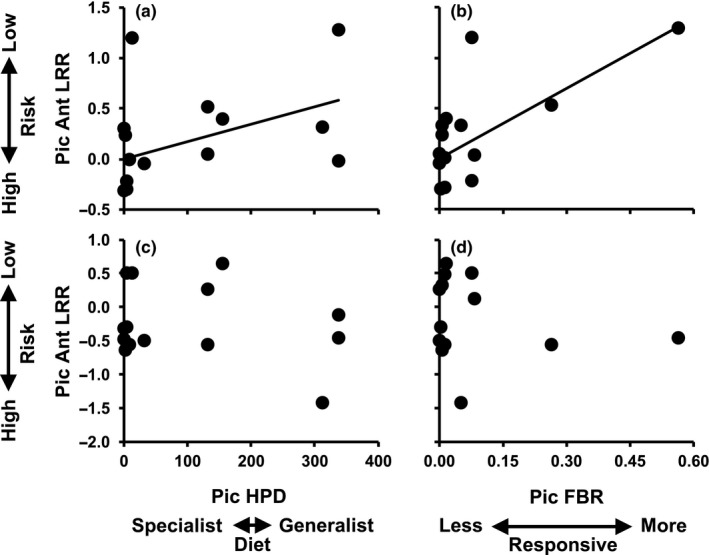
Regression plots of phylogenetically independent contrasts (PICs) based on phylogenetic tree 1, predicting the magnitude of ant predation effect [LRR_ant_: ln(control caterpillar density/ant exclusion caterpillar density)] based on (a) the diet breadth (host phylodiversity, HPD, in millions of years) and (b) the frequency of behavioral response (FBR) of 15 caterpillar species in the presence of birds (no bird exclusion). Regression plots of PICs based on phylogenetic tree 1, predicting LRR_ant_ based on (c) HPD and (d) FBR of 15 caterpillar species in the absence of birds (bird exclusion)

Bird exclusion modified the effects of caterpillar diet breadth (HPD) and behavioral responsiveness (FBR), but not of abundance, on ant predation risk (LRR_ant_) in PIC analyses; this was consistent using either tree 1 or 2 (Table 4). Specifically, positive associations between HPD and LRR_ant_ (tree 1: Adjusted *R* ^2^ = .24, *p* = .038 [Figure 2a]; tree 2: Adjusted *R* ^2^ = .15, *p* = .089 [Figure S3A]) as well as between FBR and LRR_ant_ (tree 1: Adjusted *R* ^2^ = .35, *p* = .004 [Figure 2b]; tree 2: Adjusted *R* ^2^ = .33, *p* = .005 [Figure S3B]) were found in the presence of bird effects, but no effects of HPD or FBR were found when birds were excluded (all Adjusted *R* ^2^ < .13, *p* > .1; Figure 2c,d and Figure S3C,D). These results show that broad diet breadth and the expression of behavioral defenses are associated with low ant predation risk (high LRR_ant_) only when ambient bird predation was allowed. An analysis of LRR_ant_ calculated from bird‐accessible branches only with HPD and FBR as predictors showed that caterpillar diet breadth and antipredator behavior jointly contribute to variation in ant predation risk in this context (tree 1: HPD *p* = .016, FBR *p* = .015; tree 2: HPD *p* = .051, FBR *p* = .022).

**Table 4:** ANCOVA models of ant predation risk using phylogenetically independent contrasts (picLRR_ant_) to test the influence of bird exclusion (Bird Treatment) on important predictor traits from Table 3 (Trait) based on phylogenetic trees 1 and 2 (Tree)

Trait	Source of variation	Tree	*df*	*F*	*p*
Abundance	picAbundance	1	1, 24	1.6759	.2078
		2		1.4898	.2341
	Bird treatment	1	2, 24	0.5382	.5907
		2		0.2149	.8082
	picAbundance × Bird treatment	1	1, 24	0.0155	.9020
		2		0.0752	.7863
HPD	picHPD	1	1, 24	0.0195	.8901
		2		0.0886	.7685
	Bird treatment	1	2, 24	0.3244	.7261
		2		0.1126	.8940
	picHPD × Bird treatment	1	1, 24	6.8008	.0154
		2		5.6822	.0254
FBR	picFBR	1	1, 24	1.4320	.2431
		2		0.4143	.5259
	Bird treatment	1	2, 24	0.3730	.6926
		2		0.1310	.8779
	picFBR × Bird treatment	1	1, 24	9.1393	.0059
		2		8.8869	.0065

Abbreviations: FBR, frequency of behavioral response; HPD, host phylodiversity.

### 3.3 Test of relationship between dietary specialization and behavioral defenses

As expected based on their similar effects in models accounting for bird‐exclusion effects, picFBR and picHPD were positively correlated across all 20 caterpillar species (tree 1: *t* = 2.305, *df* = 17, *p* = .034; tree 2: *t* = 2.205, *df* = 17, *p* = .042), showing that dietary generalists were more likely than specialists to respond behaviorally when attacked.

### 3.4 Test of alternative interpretation of ant predation risk

In the experiment measuring the tendency of each caterpillar species to flee from ants, dietary specialization was negatively associated with the tendency to flee from ants. That is, picFF was positively correlated with picHPD (tree 1: *t* = 2.63, *df* = 13, *p* = .021; tree 2: *t* = 2.33, *df* = 13, *p* = .036). This result is not consistent with the EFSH or with the notion that LRR_ant_ indicates escape tendency rather than predation risk.

## 4 DISCUSSION

The caterpillar community we studied exhibited an increased risk of ant predation associated with dietary specialization in the context of ambient bird predation but not in the absence of birds. This finding stands in contrast to the association between reduced attack and dietary specialization in comparative analyses of multiple caterpillar species from other communities (Bernays, 1989; Bernays & Cornelius, 1989; Dyer, 1995, 1997; Dyer & Floyd, 1993). Before addressing the apparent failure of the enemy‐free space hypothesis (EFSH) in our study, we consider some alternative interpretations of this result allowed by the experimental design used here and reported in Singer et al. (2017). Extra scrutiny is warranted because increased ant predation on dietary specialist caterpillars was not anticipated by theory or the weight of prior evidence.

There are at least two alternative scenarios consistent with the EFSH that could cause ant exclusion to increase the densities of dietary specialists more than generalists. First, we considered the possibility that nonconsumptive effects of ants (Preisser, Bolnick, & Benard, 2005) drove the patterns observed here. In this case, dietary specialist caterpillars could have fled ant‐infested areas to a greater degree than did generalists, resulting in reduced specialist caterpillar density in association with ant access to branches. If true, apparent evidence for high risk of ant predation for a caterpillar species might actually be evidence of successful escape from ants, thus supporting the EFSH prediction and previous evidence from ant–caterpillar–plant studies (Bernays, 1989; Bernays & Cornelius, 1989; Dyer, 1995, 1997; Dyer & Floyd, 1993). However, we reject this interpretation because we found a positive association between the frequency of fleeing from ants (locomotion, dropping, or rappelling on a silk line) and diet breadth (HPD) in this caterpillar community.

Second, we consider the possibility that apparent evidence against the EFSH resulted from an artifact of greater overall mobility of dietary generalist caterpillars. If ant predation were biased toward generalist caterpillars and the magnitude of biased ant predation were weaker than the effect of biased branch recolonization by more mobile generalist caterpillars, then we would expect a result similar to the one we observed. For this scenario to be true, however, the variation in mobility of caterpillar species would be a stronger predictor of LRR_ant_ than would caterpillar diet breadth (HPD). Indeed, we would expect HPD to lose its predictive power altogether in models that also include mobility. Again, however, there is no support for these predictions in our model selection analysis, in which caterpillar mobility was not an important predictor of ant predation risk (LRR_ant_).

Having discounted these alternative interpretations, we conclude that the ant predation of caterpillars does contradict the main prediction of the EFSH in the context of ambient bird predation (Singer et al., 2017). In addition to confirming the unexpected findings of Singer et al. (2017) with a more rigorous methodology, our analyses demonstrate two new patterns: antipredator behavior of caterpillar species is linked to avoidance of ant predation by dietary generalists and caterpillar species' abundance is positively associated with ant predation risk.

### 4.1 Contingency of ant predation on bird predation

The findings here confirm those of Singer et al. (2017) that ant predation is detectably biased toward dietary specialist caterpillar species in the context of ambient bird predation, but not when birds are experimentally excluded. While there are several possible explanations for this result (Singer et al., 2017), new evidence from the present study shows that behavioral defenses of caterpillars, in conjunction with diet breadth, are associated with reduced ant predation risk only in the context of bird predation. We think this new evidence points toward the second hypothesis outlined in Singer et al. (2017): that bird predation reduces the abundance of alternative arthropod predators (e.g., salticid and thomisid spiders, pentatomid and reduviid bugs), thus enhancing the role of ants as the primary invertebrate predators of caterpillars. We suspect that caterpillar behavioral defenses in response to contact, which are shown here to be effective against ants, are far less effective against these other arthropod predators because the latter typically ambush their prey (Foelix, 1996; Schuh & Slater, 1995). With the abundance of such arthropod predators greatly reduced by birds (shown to be a widespread phenomenon in Mooney et al., 2010), our experimental design can thus detect the relationship between caterpillar traits relevant to ant predation risk and changes in caterpillar density associated with ant exclusion. When bird exclusion allows for a greater influence of alternative invertebrate predators, however, their predatory effects on caterpillars might disrupt this statistical relationship. Dietary generalist caterpillars' more frequent use of behavioral defenses, such as thrashing, biting, regurgitating, dropping, and locomoting, can explain why generalists experience reduced ant predation risk compared to specialists (see Section 4.2). However, the additive effects of behavioral responses (FBR) and diet breadth (HPD) on ant predation risk (LRR_ant_) indicate that behavioral defenses are not the whole explanation for the EFS disadvantage of dietary specialization. It is likely that dietary specialist caterpillars not only lack strong behavioral defenses against ants, but also possess additional life‐history traits, such as fidelity to specific microhabitats, that make them especially vulnerable to ant predation (see Section 4.3).

### 4.2 Dietary specialization and behavioral defenses

Our results support the hypothesis that behavioral defenses associated with dietary generalization offsets or trades off with defenses typically associated with dietary specialization, such as camouflage and chemical defenses. Most previous studies testing the EFSH have used palatability assays with ecologically isolated herbivore–predator interactions (e.g., Bernays, 1989; Bernays & Cornelius, 1989; Dyer, 1995, 1997; Dyer & Floyd, 1993), and these studies show that chemical defenses can be smelled or tasted such that invertebrate predators predominantly reject dietary specialist caterpillar prey on the basis of unpalatability. However, little attention has been given to the effects of herbivore diet breadth on predation risk in the context of in situ tritrophic interactions, which allow a more inclusive set of interactions between predators and herbivores to occur on their natural host plants (Singer et al., 2014). Several other studies have noted the association between dietary generalism and greater antipredator behavioral responses in insect herbivores, especially caterpillars (Bernays, 1988; Coley et al., 2006; Vencl et al., 2005). However, the functional significance of this observation has remained elusive. Although evidence suggests that chemical unpalatability of dietary specialist herbivores supersedes the antipredator effectiveness of behavioral defenses in other communities (Dyer, 1995, 1997), our study illustrates a community in which behavioral defenses associated with dietary generalism take precedence over dietary specialization and its associated defensive traits, at least against ant predation.

As a possible explanation for the ineffective defenses of dietary specialist caterpillars against ant predation, we suggest that their chemical defenses are especially weak, at least during early larval stages when ants would be predominant predators (Remmel et al., 2011). We note the high frequency of camouflage as a visual defense strategy of caterpillars in the temperate forest community we studied (Lichter‐Marck et al., 2015). Like visual aposematism (Zvereva & Kozlov, 2016), visual camouflage is expected to be more effective against selective, visual predators (birds) than against opportunistic, nonvisual predators (ants). Therefore, the superior use of camouflage defense by dietary specialist caterpillars in this community (Singer et al., 2014) could explain why the EFSH pertains to bird predation, but not to ant predation. Although apparency versus camouflage to the human eye does not necessarily predict unpalatability versus palatability (Dyer, 1995), we hypothesize that the camouflaged dietary specialists in our system mostly lack strong chemical defenses acquired from their host plants, precluding them from possessing the unpalatability to ants observed in previous studies (Zvereva & Kozlov, 2016).

For the small set of caterpillars in this community that possess warning signals and are thus potentially aposematic toward visual predators (Lichter‐Marck et al., 2015), we suggest that the plant community we studied offers limited opportunities for dietary specialist caterpillars to acquire unpalatability toward ants. Ants in this community and others exhibit size‐dependent predation on caterpillars (Remmel et al., 2011; Singer et al., 2017), such that small caterpillars suffer the highest predation risk. The young leaves of temperate trees that host the small (early instar) caterpillars in our system have limited concentrations of small, acutely toxic secondary metabolites that can be readily sequestered by insect herbivores (Feeny, 1976) compared to toxin‐sequestering caterpillars that feed on tropical trees and herbaceous plants (Coley & Aide, 1991; Dyer & Coley, 2002) used in earlier work (Dyer, 1995, 1997; Dyer & Floyd, 1993). Therefore, the early instar caterpillars in our system might be relatively palatable to ants, regardless of their diet breadth, warning signals, and physiological ability to sequester plant allelochemicals. As the toxin‐sequestering caterpillar species grow and consume greater amounts of foliage, the dietary specialists have better opportunities to sequester allelochemicals and attain unpalatability (e.g., Boege, Agrawal, & Thaler, 2019; Dyer, 1995; Quintero & Bowers, 2018), which protects them from the relatively selective predation of birds (Lichter‐Marck et al., 2015; Singer et al., 2014). Although several authors have recently argued against the conventional wisdom that plant chemical defenses are more potent in tropical versus temperate communities (Anstett, Nunes, Baskett, & Kotanen, 2016), our argument depends on the particular properties of plant secondary metabolites that determine their utility as sequestered defenses for insect herbivores (Dyer & Coley, 2002; Opitz & Müller, 2009).

### 4.3 Implications for life‐history trade‐offs

Based on this and previous evidence (Lichter‐Marck et al., 2015; Singer et al., 2014), we propose that dietary specialization of caterpillars in this community mediates a life‐history trade‐off between (a) high behavioral plasticity (high mobility and defensive behavioral responsiveness) and high risk of bird predation for dietary generalists versus (b) low behavioral plasticity (low mobility, low behavioral responsiveness, and high behavioral stereotypy) and high risk of ant predation for dietary specialists. In effect, birds and ants function as the key predators driving a life‐history trade‐off for herbivores in the context of tritrophic interactions. While dietary specialization entails superiority in host plant‐dependent primary defenses against bird predation, such as camouflage (Lichter‐Marck et al., 2015; Singer et al., 2014) and unpalatability due to sequestration of plant toxins (e.g., Petschenka & Agrawal, 2015), the cost of dietary specialization is inferiority in secondary (behavioral) defenses against ant predation (this study). In addition, previous work on this community showed that dietary specificity among caterpillar species, whether camouflaged or apparent, was associated with fidelity to particular locations on the host plant (behavioral stereotypy: Singer et al., 2014), indicating that dietary specialist species are spatially confined with limited behavioral avoidance and escape options relative to dietary generalists. Lichter‐Marck et al. (2015) showed that behavioral stereotypy was an important predictor of reduced bird predation risk among caterpillar species in this community. We suggest that dietary specialization is generally associated with limited behavioral plasticity because host specificity may impose a high fitness cost on antipredator behaviors that make otherwise camouflaged caterpillars conspicuous to birds and force caterpillars, regardless of primary defense traits, off their host plant.

The finding that ant predation risk per caterpillar increased with a caterpillar species' abundance was the only key result that was not contingent on bird exclusion. This result suggests that ant predation efficiency increased in proportion to the rate at which ants encountered different caterpillar species, that is, density‐ or frequency‐dependent predation by ants (e.g., Kuang & Chesson, 2010; Sherratt & Harvey, 1993). Although the underlying ecological mechanism cannot be determined here, this pattern of predation has interesting ecological and evolutionary implications for the caterpillar community. Frequency‐dependent predation can be an important mechanism promoting species coexistence in the prey community (e.g., Horst & Venable, 2018; Kuang & Chesson, 2010). Furthermore, if rare caterpillar species gain some refuge from ant predation, it is possible that lepidopteran life‐history strategies entailing rarity might be favored under strong selection from ant predation.

## 5 CONCLUSIONS

The EFSH is a central idea with much potential explanatory power in the ongoing quest to understand the evolutionary ecology of dietary specialization in insect herbivores (Forister, Dyer, Singer, Stireman, & Lill, 2012; Schoonhoven, Loon, & Dicke, 2005). However, there is currently a limited understanding of the ecologically contingent roles of predator diversity and herbivore antipredator behavior in the evolutionary ecology of herbivore dietary specialization. From this study, we conclude that the role of generalist predators can be more variable and complex than Bernays and Graham (1988) originally proposed. Our results suggest that the functional diversity of predators and their interactions may select for diversity in the diet breadth of insect herbivores. This conclusion offers greater explanatory power for the observed patterns of dietary specialization in insect herbivores than could the original formulation of the EFSH. After all, it is the variation in dietary specialization within and among insect herbivore communities that requires explanation (Forister et al., 2015), not merely the preponderance of dietary specialization (Singer, 2008). Although mounting evidence suggests that tritrophic interactions involving multiple enemies, herbivores, and plants are a critical source of selection on insect herbivore diet breadth and associated life‐history traits (Vidal & Murphy, 2018), further work is needed to address mechanisms of ecological contingency among the effects of contrasting predator types, herbivore behavioral responses to multiple predators, and bottom‐up effects of plant communities.

## CONFLICT OF INTEREST

The authors declare no conflicts of interest.

## AUTHOR CONTRIBUTIONS

M.S.S., E.R.J., R.E.C., and I.H.L.‐M. collected the data; M.S.S., K.A.M., and K.D.W. analyzed the data; M.S.S. and K.D.W. wrote the first draft. All authors contributed to editing the manuscript.

## Data Availability

Table S1 provides the data needed to reproduce the analyses presented here. Additional data showing calculations of some of the data in Figure 1 and Table S1 are archived in Dryad (https://doi.org/10.5061/dryad.0k2s8k1).
