# Supplementary figures and images for: Dietary specialization is conditionally associated with increased ant predation risk in a temperate forest caterpillar community
Source: Ecol Evol. 2019 Oct 11;9(21):12099–112. doi: 10.1002/ece3.5662 (PMC6854387; doi:10.1002/ece3.5662)

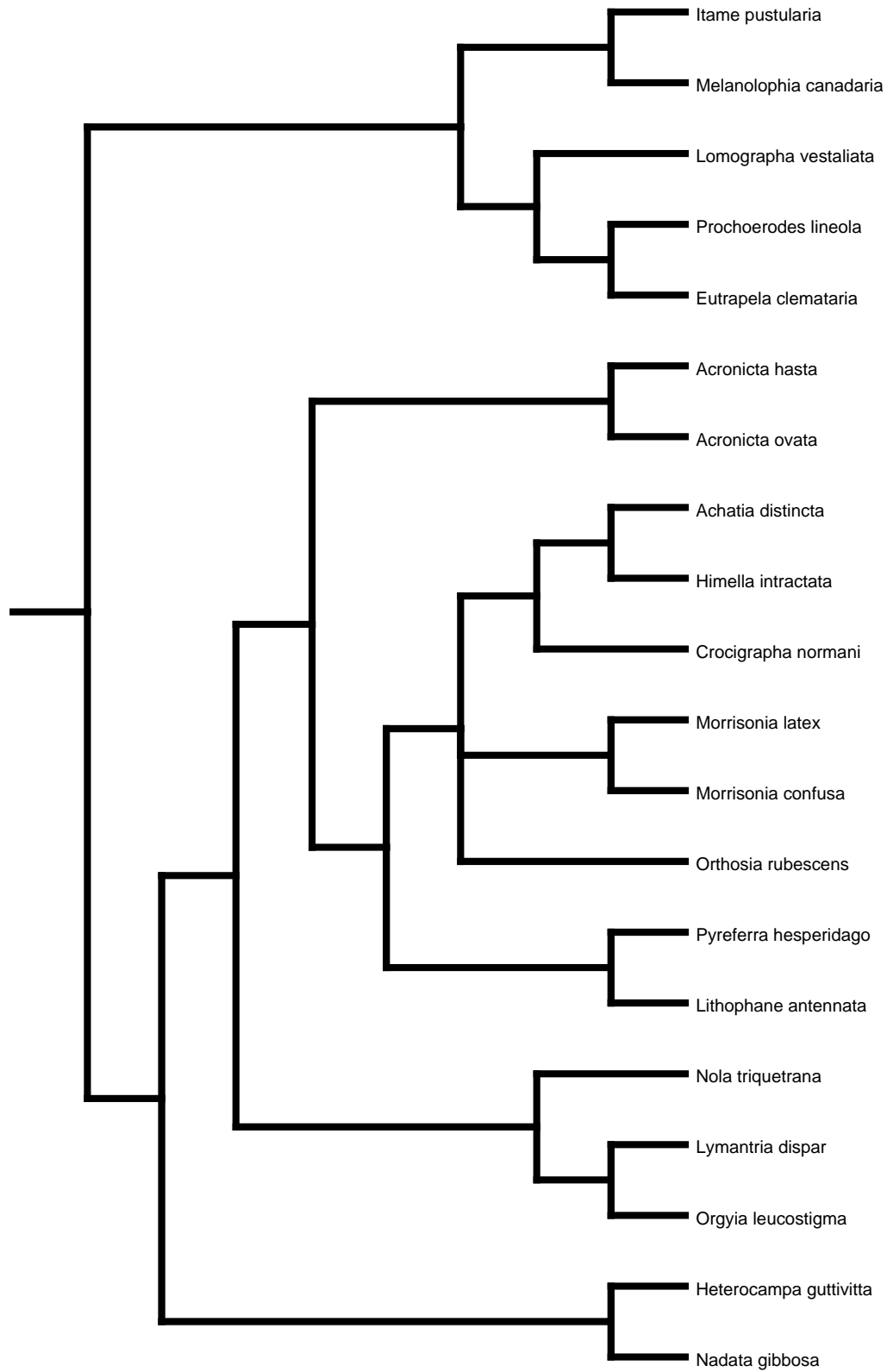

BASE++

Supplement: Supplementary file 1 [file ECE3-9-12099-s001.pdf]

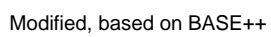

Modified, based on BASE++

Supplement: Supplementary file 2 [file ECE3-9-12099-s002.pdf]

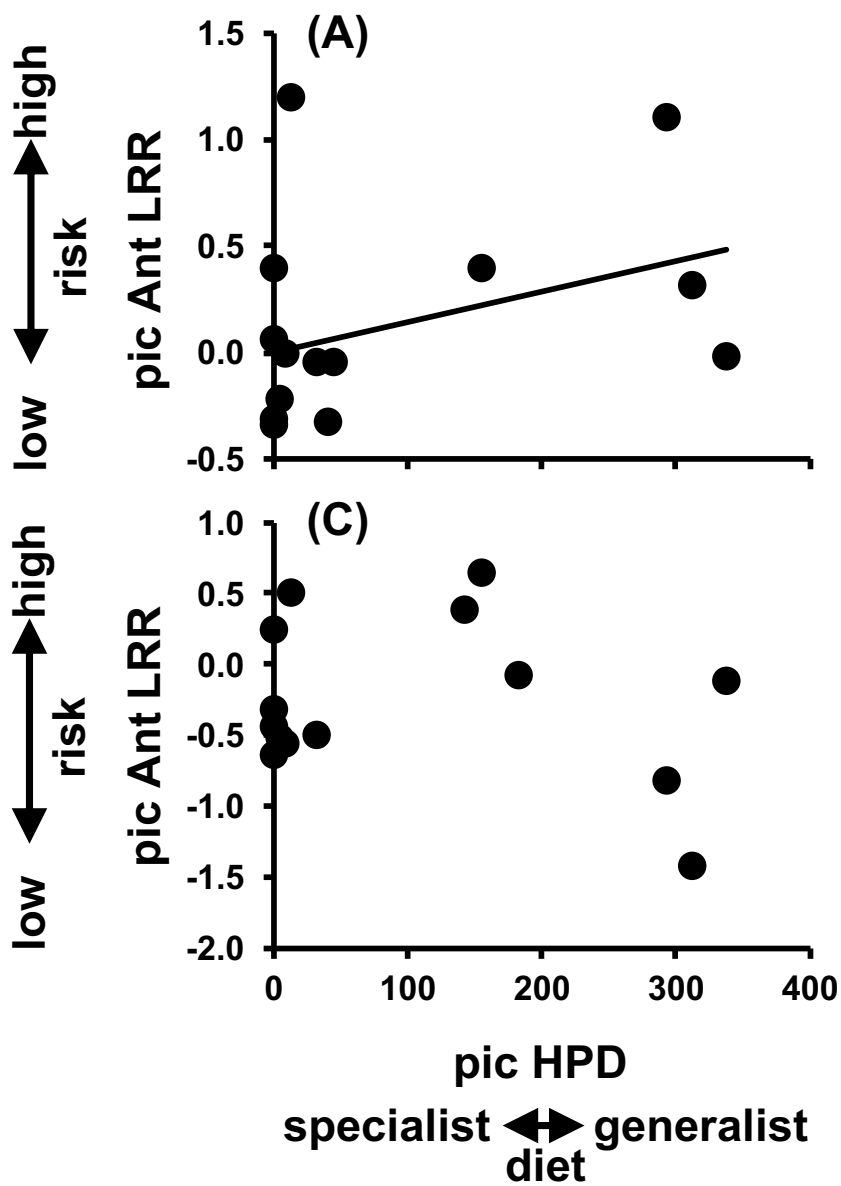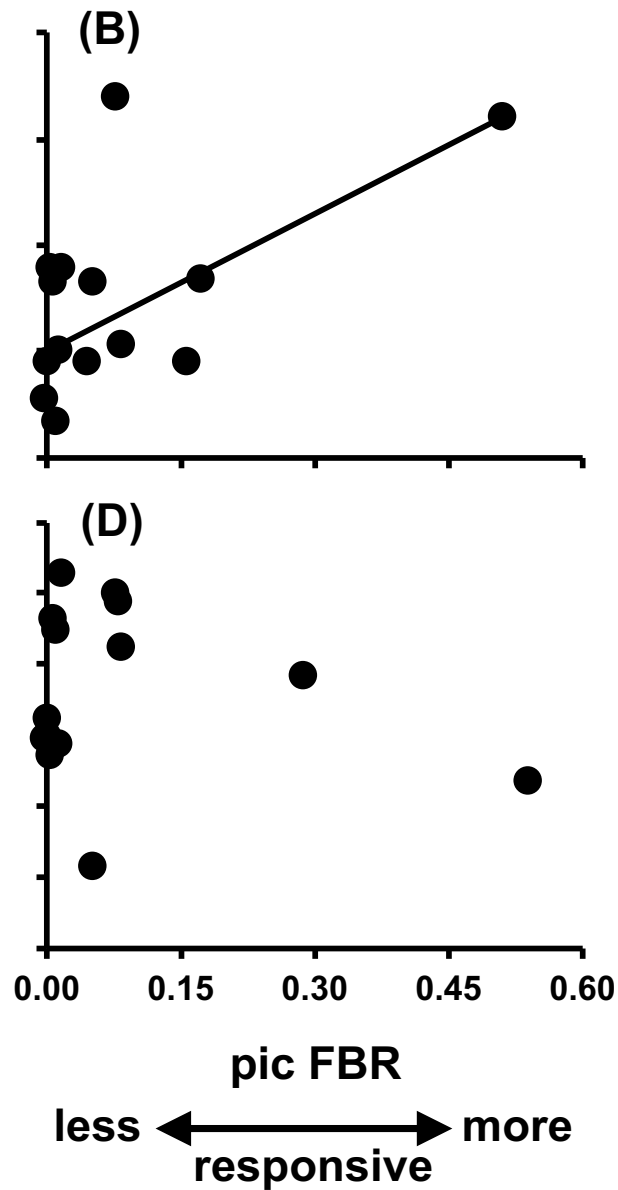

Supplement: Supplementary file 3 [file ECE3-9-12099-s003.pdf]
