# Supplementary material for: Dietary specialization is conditionally associated with increased ant predation risk in a temperate forest caterpillar community
Source: Ecol Evol. 2019 Oct 11;9(21):12099–112. doi: 10.1002/ece3.5662 (PMC6854387; doi:10.1002/ece3.5662)
Supplement: Supplementary file 4 [file ECE3-9-12099-s004.docx]

| Caterpillar species | HPD (my) | LRR_ant_ Total | LRR_ant_ unbagged | LRR_ant_ bagged | Mean length (cm) | FBR | Abundance (2011-12) | mobility | FF |
| --- | --- | --- | --- | --- | --- | --- | --- | --- | --- |
| *Achatia distincta* | 598 | -0.535 | -0.620 | -0.498 | 2.056 | 0.788 | 66 | 0.066 | 0.593 |
| *Acronicta hasta* | 121 | -0.388 | n.d. | -0.185 | 1.286 | 0.667 | 18 | 0.000 | 0.038 |
| *Acronicta ovata* | 121 | -0.205 | n.d. | -0.386 | 0.837 | 0.846 | 13 | 0.000 | 0.125 |
| *Crocigrapha normani* | 598 | 0.816 | 0.517 | 1.424 | 1.682 | 0.909 | 11 | 0.125 | n.d. |
| *Eutrapela clemataria* | 564 | -0.072 | -0.078 | -0.074 | 2.017 | 0.800 | 35 | 0.167 | 0.826 |
| *Heterocampa guttivitta* | 342 | 0.259 | -0.245 | 0.358 | 1.152 | 0.636 | 22 | 0.071 | 0.143 |
| *Himella intractata* | 552 | -0.292 | 0.091 | -0.422 | 2.265 | 0.784 | 37 | 0.043 | 0.412 |
| *Itame pustularia* | 121 | -0.289 | 0.042 | -0.292 | 1.691 | 0.849 | 232 | 0.045 | 0.826 |
| *Lithophane antennata* | 598 | -0.080 | -1.162 | 0.770 | 2.347 | 0.882 | 17 | 0.051 | 0.300 |
| *Lomographa vestaliata* | 121 | 0.354 | 1.952 | -0.522 | 1.470 | 0.722 | 18 | 0.000 | 0.909 |
| *Lymantria dispar* | 598 | 0.002 | -0.077 | 0.626 | 2.067 | 0.800 | 15 | 0.020 | 0.333 |
| *Melanolophia canadaria* | 598 | -0.234 | -0.138 | -0.333 | 1.919 | 0.729 | 277 | 0.122 | 0.889 |
| *Morrisonia confusa* | 598 | -0.246 | -0.123 | -0.386 | 1.342 | 0.877 | 114 | 0.069 | n.d. |
| *Morrisonia latex* | 598 | 0.200 | 0.334 | 0.060 | 1.179 | 0.889 | 27 | 0.125 | n.d. |
| *Nadata gibbosa* | 121 | -0.413 | -1.162 | -0.185 | 1.805 | 0.611 | 18 | 0.063 | 0.000 |
| *Nola triquetrana* | 121 | -0.568 | 0.565 | -1.184 | 0.904 | 0.000 | 28 | 0.018 | 0.100 |
| *Orgyia leucostigma* | 598 | -0.292 | n.d. | -0.232 | 1.611 | 0.872 | 47 | 0.070 | 0.000 |
| *Orthosia rubescens* | 598 | -0.094 | -0.651 | 0.300 | 2.207 | 0.860 | 43 | 0.075 | 0.611 |
| *Prochoerodes lineola* | 501 | 0.488 | n.d. | 0.508 | 2.227 | 0.929 | 14 | 0.000 | n.d. |
| *Pyreferra hesperidago* | 121 | -1.052 | n.d. | -0.878 | 2.077 | 0.591 | 22 | 0.026 | 0.038 |
